# Evaluation of efficacy of Aloe Vera as a Disinfectant by Immersion and Spray methods on Irreversible Hydrocolloid Impression Material and its Effect on the Dimensional Stability of Resultant Gypsum Cast - An in Vitro Study

**DOI:** 10.25122/jml-2019-0050

**Published:** 2019

**Authors:** Roopsi Trivedi, Rajashekar Sangur, Lakshmana Rao Bathala, Shitij Srivastava, Srishti Madhav, Preeti Chaubey

**Affiliations:** 1. Department of Prosthodontics, Sardar Patel Postgraduate Institute of Dental and Medical Sciences, Lucknow, Uttar Pradesh, India.; 2. Department of Prosthodontics, Rama Dental College Hospital and Research Centre, Kanpur, Uttar Pradesh, India.; 3. Department of Prosthodontics, Lenora Institute of Dental Sciences, Rajahmundry, Andhra Pradesh, India.; 4. Department of Prosthodontics, Sardar Patel Postgraduate Institute of Dental and Medical Sciences, Lucknow, Uttar Pradesh, India.; 5. Dental College Azamgarh, Azamgarh, Uttar Pradesh, India.

**Keywords:** Aloe vera, Cast, Dimensional Stability, Disinfectant, Irreversible Hydrocolloid

## Abstract

The need to use a natural alternative for disinfecting dental impression materials, which should be biocompatible and effective, led us to evaluate the efficacy of Aloe vera as a disinfectant by immersion and spray method on alginate impression material and its effect on the dimensional stability of the resultant gypsum cast.

The efficacy of the disinfection procedures against Staphylococcus aureus, Pseudomonas aeruginosa, and Candida albicans was evaluated by determining the number of colony-forming units (CFU) recovered after disinfection of alginate discs inoculated with 1x106 CFU for defined intervals with aloe vera solution (99.96%). Dimensional stability was determined from the mean percentage deviation of three measurements that were taken between the fixed points on the casts using a traveling microscope and compared with corresponding measurements from the master model and controls. Statistical analysis of data was determined by analysis of variance.

We found out that there is a mean percentage reduction in colony count of S. aureus, P. aeruginosa, and C. albicans after 3 minutes of immersion in aloe vera and after 3 minutes spray disinfection. Complete elimination of all the microorganism cells after 7 min immersion and spray disinfection. There was a statistically significant difference in the increase of the mean anteroposterior (AP) and cross arch (CA) dimension after 3 and 7 minutes immersion in aloe vera.

Spraying with aloe vera for 7 minutes was proved to be the most effective disinfection procedure without altering dimensional stability.

## Introduction

Irreversible hydrocolloid or alginate is the most commonly used impression material in dentistry since it is easy to manipulate, does not imply specialized equipment, and is low-priced [1]. Alginate is based on natural substances extracted from brown seaweed. As irreversible hydrocolloids are composed of 80% water, they are subject to the phenomena of imbibition and syneresis [2].

The patient’s saliva, dental plaque, and blood are a potential source of contamination for impression materials that can be a health hazard and have detrimental effects on the outcome. The control of cross-infection and lack of its standard procedures are crucial topics while dealing with dental impression materials. Irreversible hydrocolloid being partly organic, hydrophilic, irregular, and porous, ensures the retention and growth of microorganisms [3].

Henceforth, we emphasize the importance of immediate disinfection of dental materials as a standard cross-infection protocol [4] to control the transfer of infectious diseases from saliva and blood of the patient to dentists and technicians.

Disinfectants in routine use (sodium hypochlorite, glutaraldehyde) can have several deleterious effects like burning sensation of mouth and throat, irritation of eyes and skin, watering of eyes, and others. Hence, they are harmful to both health personnel and the environment [5]. Therefore, the need is to find a more natural biocompatible alternative with comparable disinfection efficacy. Aloe barbadensis Mill is a small succulent herb and has potent antibacterial, antifungal, and antiviral properties [6].

The disinfection only removes microorganisms from the surface of the impression, but an unwanted side-effect of this process is the potential for a change in the dimensions of the impression due to interaction between the set material and the disinfecting solution. Alginate is a hydrophilic material that can absorb water present in the disinfecting solution and may result in the distortion of the impression. Disinfection of alginate impressions by both spray and immersion methods have been evaluated in the past [7] with widely varying results. A general conclusion was that dimensional changes observed with disinfection were not clinically significant for most uses of irreversible hydrocolloid [8]. However, the most accurate casts were retrieved from impressions disinfected by spray rather than immersion [9,10]. Aloe vera has been used for ages for medicinal purposes and can be used fruitfully in the field of dentistry as well. The main advantage of the use of aloe vera as a disinfectant is that it is a natural product that has no or minimal side effects, readily available, less expensive, and, most importantly, is 100 percent biodegradable and does not cause any harm to the environment. As no study has been done yet to evaluate the efficacy of aloe vera as a disinfectant by immersion and spray method on an alginate impression material and its effect on the dimensional accuracy of the resultant gypsum cast, this study had been planned.

## Materials and Methods

### Sample size

Due to the paucity of data and lack of a standard formula for calculating sample size in experimental in vitro studies, the sample size in this study was modeled based on an earlier study of similar nature carried out by Rentzia A et al. [11]. A total of 40 casts were prepared from dental stone over alginate impressions made from the master model. Eight casts were kept as controls and washed with distilled water only. The remaining casts were divided equally into immersion and spray groups. Grouped casts were further subdivided into groups of 3 minutes and 7 minutes, depending upon the contact period with the disinfectant.

For the microbiological study, alginate impressions were converted into identical discs molded in a sterile 5 cc syringe measuring 10x5 mm. A total of 42 discs were prepared for each organism. For each group, 10 discs were used as control. The rest of the discs were equally allotted to four disinfection subgroups after inoculation, depending on the method and duration of disinfection.

### Laboratory procedures

#### Evaluation of antimicrobial efficacy of aloe vera

Standard strains of staphylococcus aureus (ATCC-25923), Pseudomonas aeruginosa (ATCC-27853) and Candida albicans (PTCC-5027) were used for making inoculums in test tubes containing sterile broth, each having 1x107 colony-forming unit/ml (CFU/ml). An inoculum of 0.1 ml containing 1x106 CFU (through serial dilution in nutrient broth) was applied using a sterile pipette (Astra AMP 103LX) on the surface of the alginate disc kept on the sterile Petri dish. Inoculated discs were allowed to dry in class 2 laminar flow (Kartos International) for 20 minutes. One blank disc without inoculum and one blank disc with nutrient broth acted as a negative control. Eight discs after inoculation in each group were not disinfected and acted as a positive control. The rest of the discs in each group were divided into four groups that were sterilized with aloe vera immersion or spay for 3 and 7 minutes. For the immersion group, the discs were immersed in a sterilized Petri dish containing 99.96% pure Aloe vera solution (99.96% pure, Patanjali Ayurved) for 3 and 7 minutes. For the spray group, discs were treated with aloe vera spray (3 puffs in 10 seconds) and stored in sterile plastic bags for 3 and 7 minutes.

Disinfected discs were kept aseptically in 5 ml nutrient broth in centrifuge tubes and vortexed for 1 minute, after which 1 ml aliquots were removed and kept in 1.5 ml Eppendorf tubes and centrifuged at 3000 rpm for 5 minutes (REMI R-8C) centrifuge for the recovery of bacterial cells. Following centrifugation, pellets were re-suspended in the nutrient broth that was supplemented with 0.5% sodium thiosulfate for neutralization of remaining disinfectant and were plated on blood agar for S. aureus, Mueller Hinton agar (Himedia) for Pseudomonas aeruginosa and Sabouraud dextrose agar for Candida albicans. Following inoculation, media were kept in an incubator (Surgico Industries) at 37 degrees C for 24 hours. Following incubation, colonies on media, if any, were counted under a light microscope and recorded. The number of colonies was then multiplied by 5 to calculate the number of bacterial CFU/ml.

#### Evaluation of dimensional stability

The dimensional stability of the impressions was assessed indirectly by measuring the dimensions on gypsum casts recovered from impressions of the dentate maxillary arch of a typodont (Columbia typodont model M 860). Small round holes were prepared with number 8 round bur on the mesio-palatal cusps of both the first molars and on the mesio-incisal portion of right central incisor, which served as reference marks for making anteroposterior (incisor to molar) and cross- arch (molar to molar) measurements (Figure 2).

**Figure 1:**
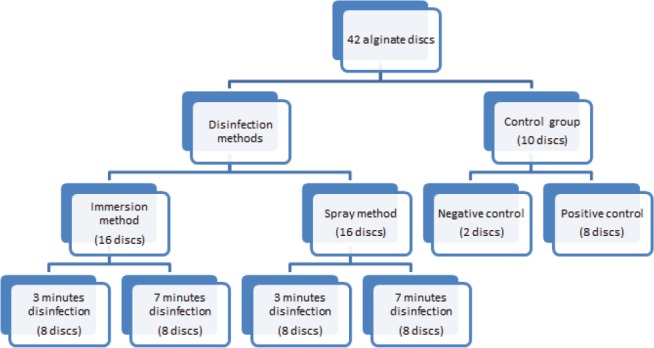
Distribution of alginate discs for disinfection.

**Figure 2:**
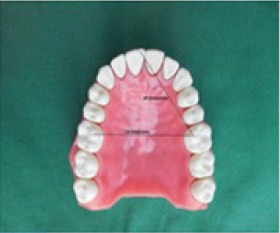
Anteroposterior and cross - arch measurements on typodont.

**Figure 3:**
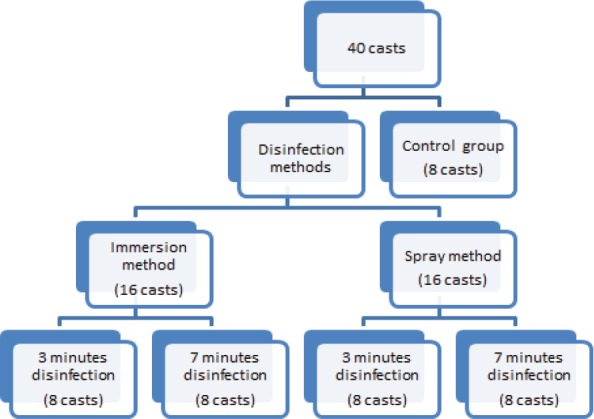
Distribution of casts for disinfection.

A total of 40 impressions were made and divided into three groups. The first group had eight impressions, which were merely washed with water and acted as a control group; the second group had sixteen impressions from which eight were disinfected by immersing in aloe vera for 3 minutes and the rest of the eight for 7 minutes. The third group also had sixteen impressions, from which eight were treated with aloe vera spray for 3 minutes and rest for 7 minutes (Figure 3).

A regular set, irreversible hydrocolloid impression material has been used in this in vitro study. Powder and water are measured according to the manufacturer’s instructions (P/W ratio 20g: 45ml), mixing with a clean, flexible bowl and a stiff-bladed curved spatula were used. The measured quantity of powder was sprinkled in the measured amount of water, and then rapid spatulation was done, followed by loading in a sterilized, maxillary perforated stock metal tray. Impressions were made 1 minute from the start of mixing and were allowed to set on the master model for 5 minutes. After removal, all the impressions were rinsed for 10 seconds with distilled water to simulate saliva removal. Test impressions were immersed or sprayed in the aloe vera at room temperature for 3 and 7 minutes, with the control group undergoing no disinfection procedure. For the immersion group, the impressions were immersed in a sterilized glass beaker containing 500 ml of 99.96% pure Aloe vera solution (99.96% pure, Patanjali Ayurved) for 3 and 7 minutes. For the spray group, impressions were treated with aloe vera spray (99.96% pure, Patanjali Ayurved) - 10 puffs in 15 seconds and stored in sterile plastic bags for 3 and 7 minutes.

After disinfection, all the impressions were again rinsed with distilled water for 10 seconds. All the impressions were cast using dental stone. A water powder ratio of 30 ml/100 gm was vacuum-mixed for 60 seconds and poured into the impressions using vibrations. The casts were allowed to sit for 45 min before separation from the impression, and none of the casts were mechanically trimmed. The casts were stored at room temperature for 24 hours prior to analysis. Cross-arch (molar to molar) and anteroposterior (incisor to molar) dimensions (mm) were measured on each cast using a traveling microscope (VKSI 0010) with an accuracy of 0.01 mm. Dimensions of each cast were taken thrice by two trained personnel separately to minimize subjective errors.

### Statistical analysis

All the data from the experimentation was entered in an Excel file. Epi-Info software (version 7) was used for data analyses. The normal distribution of continuous variables was ensured using the Konglomorov-Smirnov test. For assessment of antimicrobial efficacy, log values of colony counts were used and compared amongst intervention groups using the unpaired t-test. For dimensional accuracy, a comparison of mean values of anteroposterior and cross-arch diameter was made in the control, 3 and 7 minutes disinfection groups using ANOVA.

## Results

### Antimicrobial efficacy

Mean log values of colony counts of organisms in control group and post disinfection (after 3 min immersion & spray and 7 minutes immersion and spray) for S. aureus, P. aeruginosa, and C. albicans are depicted in table 1, 2 and 3, respectively. However, the duration of the application did result in a reduction of bacterial counts, but it was not statistically significant. Further repetitions of the study are needed on a larger scale.

**Table 1:** Comparison of colony count and log values of Staph. aureus in control discs and discs disinfected in Aloe vera.

Disc nomenclature	Mean CFU/ml (S.D.)	Mean log value of CFU (S.D.)	Mean log reduction	Mean percentage reduction (1-1x10^-log reduction^)x100 [%]	p value
**Control disc (A)**	74625.00 (28399.88)	4.93(0.07)	NA	NA	Ref.
**3 minutes spray (B)**	212.5 (99.61)	2.28(0.21)	2.65	99.77	<0.0001 (ANOVA) (A vs B vs C ) 0.351 (B vs C) (student ‘t’ test using log values
**3 minutes immersion (C)**	204.38(116.42)	2.25(0.06)	2.68	99.79	
**7 minutes spray (D)**	0.00(0.00)	NA	NA	100.00	
**7 minutes immersion (E)**	0.00(0.00)	NA	NA	100.00	

**Table 2:** Comparison of colony count and log values of pseudomonas aeruginosa in control discs and discs disinfected in Aloe vera.

Disc nomenclature	Mean CFU/ml (S.D.)	Mean log value of CFU (S.D.)	Mean log reduction	Mean percentage reduction (1-1x10^-log reduction^)x100 [%]	p value
**Control disc (A)**	78750.00 (14179.97)	4.89(0.08)	NA	NA	Ref.
**3 minutes spray (B)**	247.5 (128.70)	2.35(0.25)	2.54	99.71	<0.0001 (ANOVA) (A vs B vs C ) 0.433 (B vs C) (student ‘t’ test using log values
**3 minutes immersion (C)**	241.88(128.62)	2.33(0.23)	2.55	99.72	
**7 minutes spray (D)**	0.00(0.00)	NA	NA	100.00	
**7 minutes immersion (E)**	0.00(0.00)	NA	NA	100.00	

**Table 3:** Comparison of colony count and log values of candida albicans in control discs and discs disinfected in aloevera.

Disc nomenclature	Mean CFU/ml (S.D.)	Mean log value of CFU (S.D.)	Mean log reduction	Mean percentage reduction (1-1x10^-log reduction^)x100 [%]	p value
**Control disc (A)**	82375.00 (13627.05)	4.91(0.07)	NA	NA	Ref.
**3 minutes spray (B)**	268.75 (138.40)	2.37(0.25)	2.52	99.72	<0.0001 (ANOVA) (A vs B vs C ) 0.268 (B vs C) (student ‘t’ test using log values
**3 minutes immersion (C)**	273.75 (123.97)	2.39(0.21)	2.54	99.69	
**7 minutes spray (D)**	0.00(0.00)	NA	NA	100.00	
**7 minutes immersion (E)**	0.00(0.00)	NA	NA	100.00	

### Spray and immersion methods for 7 minutes

For all the organisms tested, no growth of any tested organism was observed in a disc sterilized for 7 minutes using either spray or immersion methods. 

### Spray method for 3 minutes

Discs disinfected for 3 minutes using the spray method showed a mean percentage reduction [(1-1x10-log reduction) x 100] of 99.77%, 99.71%, and 99.69% in colony count of S. aureus, P. aeruginosa, and C. albicans, respectively.

### Immersion method for 3 minutes

Following 3 minutes disinfection by immersion method, there is a mean percentage reduction [(1-1x10-log reduction) x 100] of 99.79%, 99.72%, and 99.72% in colony count of S. aureus, P. aeruginosa, and C. albicans, respectively.

### Dimensional Stability

**Figure 4:**
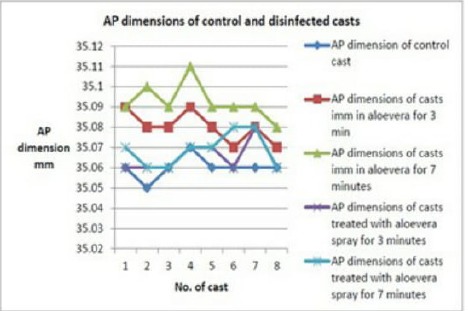
AP dimensions of control and disinfected casts

**Figure 5:**
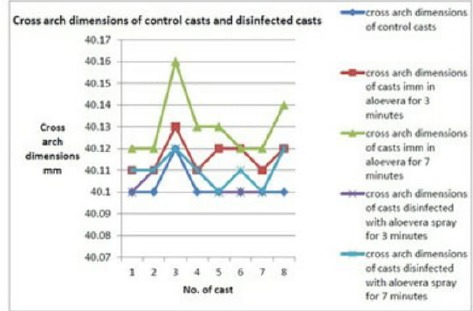
Cross arch dimensions of control and disinfected casts

Casts poured after immersion in aloe vera solution depicted a mean increase in the anteroposterior dimension of 0.01 mm, i.e., 10 microns at 3 minutes and 0.03 mm, i.e., 30 microns at 7 minutes, respectively. The difference was statistically significant (p-value by ANOVA was <0.001) (Figures 4 and 5).

There was no increase in the mean anteroposterior dimensions of casts whose impressions were kept in contact with aloe vera spray for 3 minutes. However, casts whose impressions were kept in contact for 7 minutes show a mean increase in the mean anteroposterior dimensions of 0.01 mm 10 microns, but the difference was statistically insignificant ( p-value by ANOVA was 0.28). 

The increases in the mean cross-arch dimensions of casts after 3 and 7 minutes immersion periods were 0.01 mm (10 microns) and 0.02 mm (20 microns), respectively, as compared to control casts. The difference was statistically significant (p-value by ANOVA <0.001). 

There was no increase in the mean cross arch dimensions after aloe vera spray with 3 minute contact period. However, after 7 minutes, the increase in the mean dimensions was 0.01 mm (10 microns). However, the difference was statistically insignificant (p-value 0.177).

## Discussion

Dentists, patients, and dental technicians get exposed to various types of pathogenic microorganisms through infected impression trays, materials, prostheses, and casts [12]. The most frequently recognized opportunistic pathogens are Streptococcus, Staphylococcus, Escherichia coli, Actinomyces, Antitratus, Pseudomonas, Klebsiella pneumonia, and Candida species [13]. Candida causes common opportunistic infections known as oral candidiasis found in patients with immune deficiency [14]. Pseudomonas aeruginosa is commonly associated with hospital-acquired infections and frequently found on dental instruments [15].

Oral infections are polymicrobial. The efficacy of the disinfectants against biofilms is different than against the planktonic state. Unlike planktonic cells, for which several well-defined standards have been published, fewer standard methods are available to evaluate the susceptibility of biofilm cells to disinfectants [16].

Various recent methods to disinfect dental impressions have been introduced like ozonated water [17], UV light [18], ethylene oxide gas autoclave [19], and radiofrequency glow discharge [20]. However, the efficacy of a range of disinfectants in achieving the goal is still questionable. The routinely used disinfectants such as sodium hypochlorite and glutaraldehyde, have several deleterious effects on both health and the environment. Glutaraldehyde is an irritant and can cause acute sensitivities [21], can be absorbed by inhalation, ingestion, and through the skin. Sodium hypochlorite, if it comes in contact with the eyes, causes pain, severe burning, erythema, and excessive watering. The outer layer of the cornea can also get eroded [22].

In this study, for the first time, the antimicrobial effect of Aloe vera on impression materials was investigated. Aloe barbadensis Miller is a succulent plant that resembles cactus, with green fleshy, spiny, and marginated leaves, filled with a clear, viscous gel [23]. Aloe vera has potent antibacterial, antifungal, and antiviral properties [24,25,26]. According to Zimmerman and Sims, cited in The Essential Aloe vera [27-29], Aloe vera inhibits the growth of S.aureus, S.viridans, Candida albicans, and Cornybacteria xerosis. Carrington laboratory Inc (Aloe vera in Dentistry, 1991) [30] stated that ACEMANNAN stimulates macrophages, and it also has antiviral properties. The main advantage of the use of aloe vera as a disinfectant is that it is a natural product that has no or minimal side effects, readily available, and, most importantly, is 100 percent biodegradable and does not cause any harm to the environment. In some sensitive individuals, it may cause allergic reactions, rashes, burning sensations, and rarely generalized dermatitis [28]. It was found that Aloe vera has a very potent antimicrobial action. Following 3 minutes disinfection by immersion and spray method, the percentage reduction in microbial load is more than 99%, and there is a 100% reduction after 7 minutes. This antibacterial action of aloe vera is identified due to the presence of compounds like p-coumaric acid, ascorbic acid, pyrocatechol, and cinnamic acid [31].

Alginate is derived from anhydro [beta]-D mannuronic acid or alginic acid, which is extracted from brown seaweed [32]. Since alginate is an organic hydrocolloid impression material that is hydrophilic and porous, it provides for retention and growth of microorganisms. As it is composed of 80% of water, it may subject to the phenomena of imbibition and syneresis [33]. These properties affect the dimensions of the impression during disinfection and make the time of immersion a critical factor. According to Taylor et al., synergies-associated shrinkage can be overcome by ten-minute imbibition [34]. The spraying method, on the other hand, limits the exposure of impression to the wet environment and hence maintains dimensions of impression [30] and produce accurate casts [35]. However, spraying is done with the reduced contact time, which may limit the effectiveness of disinfection, particularly for the porous hydrophilic hydrocolloids, where microorganisms can infiltrate and survive in the impression [36].

In the present study, casts immersed in aloe vera solution depicted a mean increase in the anteroposterior and cross-arch dimensions, but there was no increase in the mean anteroposterior and cross-arch dimensions of casts kept in contact with aloe vera spray.

Rueggeberg Frederick et al. [37], in their study on efficacy of sodium hypochlorite as disinfectant on irreversible hydrocolloid, state that the spray disinfection of an alginate impression did not cause dimensional differences of the poured stone casts, but the immersion disinfection created dimensional distortion of the anterior, posterior, and inter-arch model segments, which were consistent with the results of this study.

### Limitations of the study

These results cannot be absolutely extrapolated across other brands of related materials because of the possibility of minor changes in chemistry, causing significantly different responses.

Viruses were not considered for this experiment because of the potential danger in its manipulation and the inexistence of the required equipment.

The effect of remnants of aloe vera disinfectant on the resultant gypsum cast was not evaluated.

## Conclusions

1. The effectiveness of aloe vera as a disinfectant was demonstrated on three different microorganisms, namely Staphylococcus aureus, Pseudomonas aeruginosa, and Candida albicans, as the percentage reduction of the microbial load was greater than 99% after 3 minutes of disinfection by immersion and spray method and 100% reduction after 7 minutes.

2. Although there was a small difference regarding colony reduction between the two methods, i.e., immersion and spray methods at 3 minutes, this was statistically insignificant.

3. Casts whose impression was immersed in the aloe vera solution showed a mean increase in the anteroposterior dimension both at 3 and 7 minutes, and the difference was statistically significant.

4. The mean increase in the anteroposterior dimension after disinfection with aloe vera spray for 3 minutes and after 7 minutes was statistically insignificant.

5. There was a statistically significant difference in the increase of the mean cross-arch dimension after 3 and 7 minutes immersion in aloe vera.

6. The mean increase in cross-arch dimension after disinfection with aloe vera spray for 3 minutes and after 7 minutes spray was statistically insignificant.

## Conflict of Interest

The authors confirm that there are no conflicts of interest.
